# CD-TrGNN: A Complex-Domain Transformer–Graph Neural Network for ISAR Space Target Attitude Estimation

**DOI:** 10.3390/s26154705

**Published:** 2026-07-24

**Authors:** Yonghua He, Jiahao Wang, Aoxiang Pan, Wei Qu, Weigang Zhu, Yonggang Li, Wenhang Ji

**Affiliations:** Space Engineering University, Beijing 101400, China; heyonghua1980@hgd.edu.cn (Y.H.); pax@hgd.edu.cn (A.P.); quwei@hgd.edu.cn (W.Q.); zwg@hgd.edu.cn (W.Z.); liyonggang@hgd.edu.cn (Y.L.); jijiji0908@hgd.edu.cn (W.J.)

**Keywords:** ISAR, space target, attitude estimation, complex-domain deep learning, transformer, graph neural network

## Abstract

**Highlights:**

**What are the main findings?**

- CD-TrGNN performs global self-attention and topology reasoning directly in the complex domain, preserving both amplitude and phase information and substantially improving attitude estimation accuracy.
- By modeling satellite components as graph nodes with learnable adjacency, the framework captures part-level structural dependencies overlooked by conventional CNNs and vision Transformers.

**What are the implications of the main findings?**

- Complex-domain attention combined with adaptive topological reasoning offers a new paradigm for fully exploiting amplitude and phase in ISAR images, advancing space target attitude estimation.
- The end-to-end architecture directly regresses three-axis attitude angles from complex images, providing a robust and scalable solution for all-weather space surveillance and on-orbit servicing.

**Abstract:**

In ground-based space surveillance, space target attitude estimation is critical for space situational awareness, yet existing methods based on inverse synthetic aperture radar (ISAR) images suffer from three core limitations: phase information is discarded in amplitude-only processing, convolutional neural networks have a restricted global receptive field, and the physical topology of satellite components is not explicitly modeled. To address these issues, we propose a complex-domain Transformer–graph neural network (CD-TrGNN) that unifies global context modeling and adaptive topological reasoning in an end-to-end framework. Specifically, a complex-domain Transformer module (CD-Transformer) with tailored attention captures long-range dependencies among image patches while preserving both amplitude and phase information; a complex-domain graph convolution module (CD-GC) with learnable adjacency matrices and a dual-path update mechanism explicitly encodes the structural relationships among satellite parts. On a self-built ISAR complex image dataset, CD-TrGNN achieves a three-axis mean absolute error of only 1.70°, substantially outperforming six representative baselines. Ablation experiments confirm the effectiveness of complex-domain processing, global attention, and topological reasoning. At a 5 dB signal-to-noise ratio, the error remains at 2.81°, and the accuracy stays below 2° for two different satellite structures. These results demonstrate that CD-TrGNN can fully exploit the information in ISAR complex images, enabling high-accuracy and highly robust attitude estimation.

## 1. Introduction

Outer space, as a global strategic high ground, has become a key domain of great-power competition. With the rapid development of space technology, the number of space objects is growing explosively. Accurately obtaining the on-orbit attitude of non-cooperative targets—namely the pitch, yaw, and roll angles—is of great significance for space situational awareness, orbit prediction of failed satellites, space debris collision avoidance, and mission assessment of Earth-observation satellites [1,2,3]. Traditional attitude estimation methods mostly rely on optical images, employing template matching or feature point extraction; however, they are susceptible to illumination conditions and cannot meet the demands of all-time and all-weather surveillance [4,5]. For ground-based radar systems, inverse synthetic aperture radar (ISAR), with its high resolution and all-time, all-weather imaging capability, can produce fine two-dimensional images of space targets, thus providing a unique data source for satellite attitude estimation [6,7].

Unlike conventional optical images, ISAR images are intrinsically complex-domain signals, containing both amplitude information that reflects scattering intensity and phase information that represents the phase delay of electromagnetic waves [8]. However, most existing ISAR-image-based attitude estimation methods only utilize the amplitude images, discarding the phase component directly after taking the modulus of the complex data, which results in insufficient information exploitation and a bottleneck in estimation accuracy.

Existing ISAR satellite attitude estimation methods can be categorized into three major classes: template matching, three-dimensional reconstruction, and deep learning. Early studies mainly adopted template matching, whose core idea is to use a simulation database and infer the attitude by searching for the sample that best matches the observed data through similarity comparison. According to the type of matching object, these methods can be further divided into three groups: those based on radar cross-section (RCS) [9], those based on high-resolution range profile (HRRP) [10], and those based on ISAR images [11,12]. RCS matching has clear physical meaning and requires a small amount of data, but it is extremely sensitive to attitude and angular variations. HRRP matching offers relatively high real-time performance, yet it also suffers from high attitude sensitivity and weak anti-noise capability. ISAR image matching has the advantage of rich two-dimensional features, but data acquisition is complex, and the method is sensitive to noise and occlusion. In general, template matching methods are limited by the expressive power of hand-crafted features and the completeness of the database, making them difficult to meet the high-precision requirements in complex scenarios.

To overcome the limitations of template matching, researchers introduced the idea of three-dimensional reconstruction [13,14], i.e., recovering the three-dimensional structure of the target by extracting scattering center parameters and then solving the attitude. This class of methods mainly includes two technical routes. The first is the interferometric phase method [15,16,17,18], which exploits phase differences among multiple channels to construct an interference pattern and obtain depth information. It can achieve high-precision non-contact measurement, but phase unwrapping is complicated, and the environmental requirements are stringent. The second route is the sequential-image-based method [19,20,21,22], which extracts geometric and motion information from multi-view or time-series ISAR images. For example, the factorization method constructs the trajectory matrix of scattering centers and recovers the three-dimensional coordinates via singular value decomposition; it is computationally efficient but sensitive to noise and demands rigorous scattering center association. Other sequential-image methods exploit projection geometry relationships under different viewing angles for reconstruction; they offer all-weather operational capability but have relatively limited resolution and complex data processing. Although three-dimensional reconstruction methods can provide intuitive geometric constraints, their high computational complexity and susceptibility to factors such as scattering center anisotropy, trajectory occlusion, and the need for large angular data make them difficult to apply in real-time scenarios.

In recent years, deep learning techniques [23] have brought new breakthroughs to this field, and data-driven end-to-end frameworks are gradually becoming the mainstream. Existing deep learning methods can be divided into two-stage methods and end-to-end methods. Two-stage methods [24,25] first employ a convolutional neural network (CNN) to perform feature extraction or semantic segmentation on ISAR images to predict the 2D image coordinates of the satellite’s key components, and then solve the three-dimensional attitude through the PnP algorithm [26] or geometric constraint optimization. For instance, Ren Xiaolin [27] utilized Pix2pixGAN to segment the components in ISAR image sequences, formulated the attitude parameter solution as an unconstrained optimization problem, and employed particle swarm optimization to search for the optimal attitude, achieving estimation errors within 2° for both the main body and the solar panel. Li et al. [28] proposed a multi-view ISAR image sequence selection criterion based on the normal vector of the imaging plane, extracted target feature points using HRNet, and then solved the attitude optimization problem with PSO. However, such methods rely heavily on the feature extraction quality of the first stage, where intermediate errors easily accumulate, and the implementation is complex and computationally expensive. End-to-end methods [29,30] directly output the three-axis attitude angles from ISAR images through a CNN regression network, which simplifies the processing pipeline and offers the advantage of global optimization. Fan Lei et al. [31] established a regression network framework suitable for attitude estimation, where the mean attitude error of a single image can be controlled within 3.5°. Nevertheless, these methods are highly dependent on the quality and quantity of training data, and the inherent local receptive field of CNNs restricts their ability to model global spatial structures.

Notably, the above deep learning methods still face three core problems when dealing with ISAR complex images and the physical structural characteristics of satellites. First is the loss of phase information. Existing CNN and Transformer architectures are designed for real-domain images; their convolution and self-attention operations cannot directly process complex-domain data [32]. Research has shown that converting complex data into real-domain inputs significantly reduces the information representation capability of the model, causing the geometric and material features contained in the phase to be discarded. Second is limited global dependency modeling. The local receptive field of CNNs makes them ineffective at capturing the spatial dependencies between distant components such as solar panels and antennas. Although the standard Vision Transformer can model long-range dependencies, its self-attention mechanism is based on real-domain dot-product operations: if complex data are forcibly input, the operation is incompatible; if only magnitude images are fed in, it falls back into the dilemma of information loss [33]. Third is the absence of satellite topological priors. A satellite is a typical composite body consisting of multiple components such as the main body, solar panels, and antennas; the relative spatial positions and connection relationships among these components constitute important topological constraints for attitude estimation. Existing end-to-end networks usually treat the image as an unstructured grid and fail to explicitly exploit such component-level topological associations. Graph neural networks (GNNs) can model structural dependencies among nodes; however, existing GNN designs are also oriented toward real-domain features, with pre-fixed adjacency matrices that lack the adaptive ability to cope with dynamic changes in connection relationships [34].

To address the above issues, this paper proposes a complex-domain Transformer–graph neural network (CD-TrGNN), which unifies the long-range dependency modeling capability of Transformers with the structural topological reasoning capability of graph neural networks in the complex domain, constructing an end-to-end attitude estimation framework that operates in the complex domain. Specifically, CD-TrGNN builds global contextual associations among image regions through a complex-domain Transformer module, solving the problems of the limited local receptive field of traditional CNNs and the incompatibility of real-domain Transformers with complex operations. Furthermore, a complex-domain graph convolution network is employed to explicitly encode the spatial topological relationships among satellite components, adaptively learning component-level feature interactions and compensating for the insufficient exploitation of structural priors in existing methods. Through the synergy of complex-domain global perception and topological reasoning, CD-TrGNN is able to achieve high-precision and highly robust three-axis attitude estimation of space targets.

The main contributions of this paper can be summarized as follows:

(1) An end-to-end attitude estimation framework, CD-TrGNN, working in the complex domain is proposed, which combines a complex Transformer with complex graph convolution to fully exploit information in ISAR complex images.
(2) A complex-domain Transformer module is designed, which effectively captures global spatial dependencies among image patches through complex self-attention and a parallel fully connected branch.
(3) A complex-domain graph convolution module is designed, which introduces a learnable adjacency matrix and a dual-path update strategy to explicitly model the adaptive topological relationships among space target components, thereby enhancing the physical interpretability and estimation accuracy of the model.

The remainder of this paper is organized as follows: Section 2 establishes the ISAR imaging projection model for space targets and describes the dataset construction method; Section 3 presents the network architecture and core modules of the proposed CD-TrGNN in detail; Section 4 provides the experimental setup and result analysis, verifying the method’s performance through comparative experiments, ablation experiments, robustness experiments, and generalization experiments; and Section 5 concludes the paper and discusses future research directions.

## 2. ISAR Imaging Projection Model and Dataset Construction

**Figure 1:**
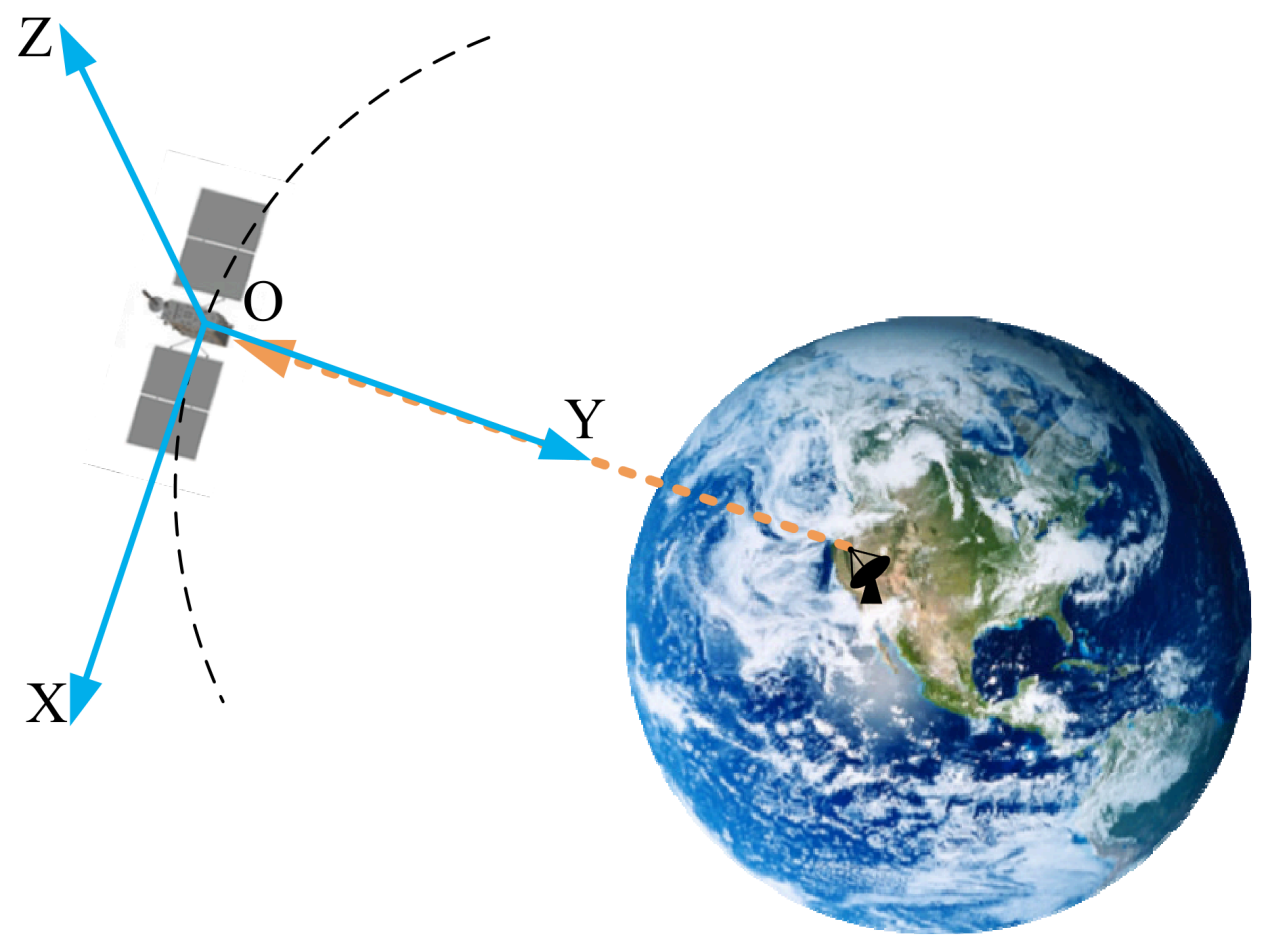
ISAR imaging geometry.

To obtain high-resolution ISAR images of a space target, a ground-based radar transmits a wideband linear frequency modulation (LFM) signal and continuously tracks the target [35]. As the target moves along its orbit, the relative motion between the target and the radar induces time-varying Doppler shifts. Through coherent processing (i.e., Fourier transform along the slow-time dimension), scattering centers with different radial velocities are resolved into distinct Doppler frequency bins, which correspond to different cross-range positions. This process provides the cross-range resolution. Combined with the range resolution achieved by the wideband signal, the two-dimensional ISAR image is formed [36]. The imaging geometry is illustrated in Figure 1. A right-handed Cartesian coordinate system is defined with the origin fixed at the target’s center of mass: the X-axis is aligned with the along-track direction of the target, the Y-axis points opposite to the radar line of sight (LOS), and the Z-axis is perpendicular to the XOY plane, completing the right-handed frame. This coordinate choice naturally associates the range direction with the Y-axis and the cross-range direction with the X-axis, facilitating the subsequent attitude analysis. In this system, the three-axis attitude angles are defined as follows: pitch is the rotation angle around the X-axis, yaw is the rotation angle around the Y-axis, and roll is the rotation angle around the Z-axis. These angles represent the orientation of the satellite body with respect to the orbital reference, and their changes are directly reflected in the projected positions and shapes of the satellite’s scattering structures in the ISAR images.

Based on the above definitions, the three-axis attitude angles of the space target are rotated within the range of [−30°, +30°] and sampled at 6° intervals, yielding 1331 images that constitute the training set. For the test set and validation set, the attitude angles are sampled at 10° intervals within the non-overlapping ranges [−23°, +27°] and [−27°, +23°], respectively, each producing 216 images. This sampling strategy guarantees sufficient coverage of the attitude space while strictly separating the data used for training and evaluation. All simulated images are generated using the standard range-Doppler (RD) imaging algorithm, which transforms the radar echoes into focused two-dimensional complex images by exploiting the range delay and Doppler shift. The parameters of the ISAR system are provided in Table 1.

**Figure 2:**
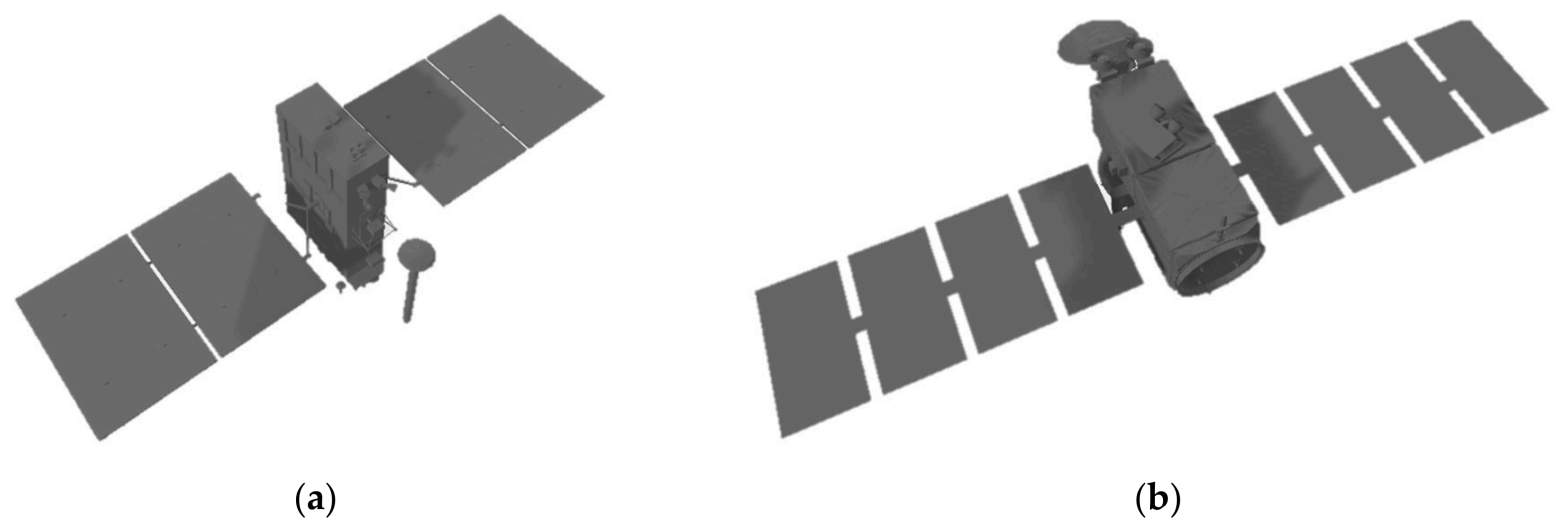
Three-dimensional models of the two space targets: (**a**) Target A; (**b**) Target B.

Based on the above simulation parameters, RD imaging is carried out for two types of space targets across a set of attitude angles uniformly sampled within the ranges defined in Section 2. This process generates a series of complex-domain ISAR images, each preserving the full amplitude and phase information of the radar echoes. The three-dimensional structural models of the two space targets are depicted in Figure 2.

**Figure 3:**
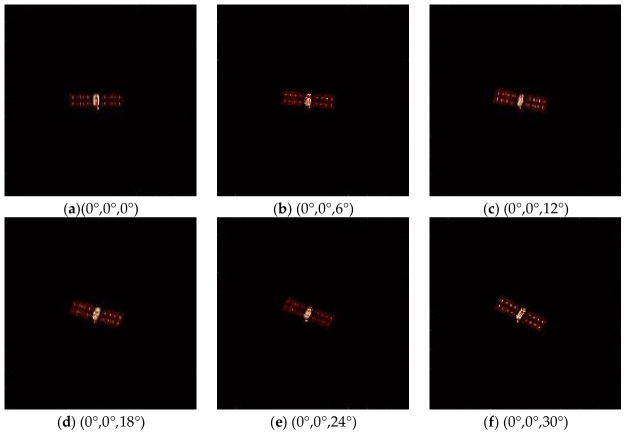
Representative ISAR magnitude images of Target A under typical attitudes with corresponding attitude angle annotations.

**Figure 4:**
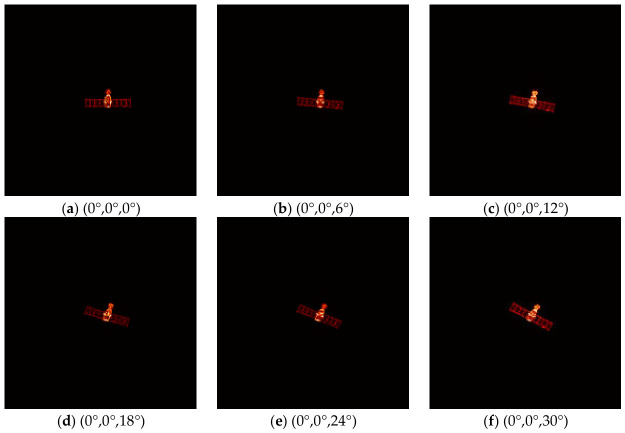
Representative ISAR magnitude images of Target B under typical attitudes with corresponding attitude angle annotations.

Since complex images are not convenient for intuitively demonstrating attitude variations, we visualize their magnitude counterparts, as shown in Figure 3 and Figure 4. Specifically, Figure 3 displays the ISAR imaging results of Target A under representative attitudes, while Figure 4 presents those of Target B. Both figures clearly illustrate how the scattering structures of key components—such as the satellite main body and solar panels—vary with attitude changes in the projection plane.

## 3. Model Construction

To address the three core issues of existing ISAR-based space target attitude estimation methods—namely insufficient exploitation of complex information, limited global spatial dependency modeling, and the absence of explicit satellite topological priors—this paper proposes CD-TrGNN, a complex-domain Transformer–graph neural network that unifies global context modeling and adaptive topological reasoning. This section elaborates on the architectural design and implementation details of the proposed network: Section 3.1 presents the overall network architecture and the end-to-end training strategy; Section 3.2 describes the core design of the CD-Transformer module, which is responsible for long-range dependency capture; and Section 3.3 details the topological modeling approach of the CD-GC module, which explicitly encodes part-level structural relationships.

### 3.1. Overall Network Architecture

**Figure 5:**
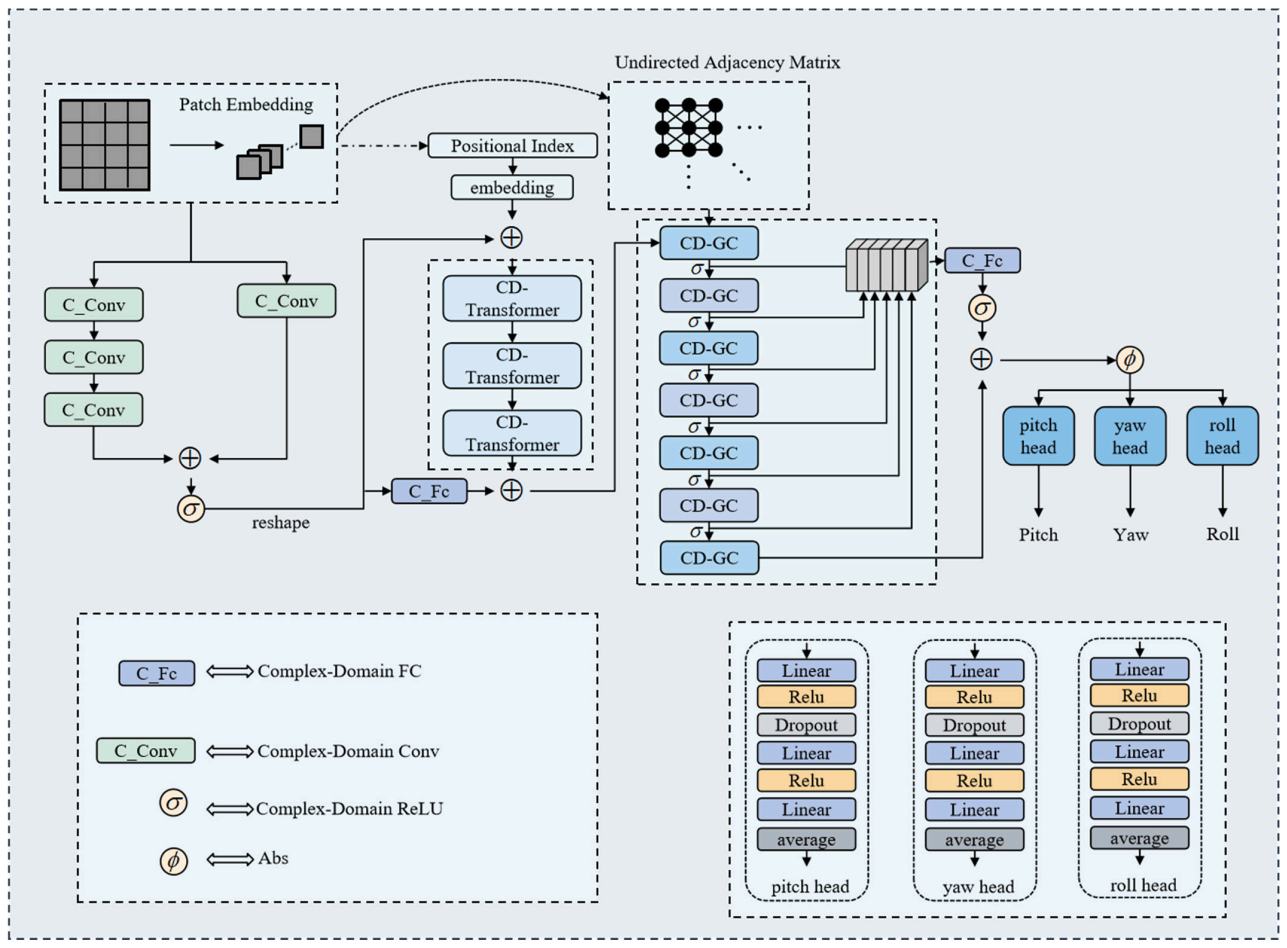
CD-TrGNN network structure.

CD-TrGNN accomplishes the end-to-end mapping from an ISAR image to three-axis attitude angles entirely in the complex domain. The overall architecture, as shown in Figure 5, consists of four main stages: complex convolutional feature extraction, CD-Transformer global dependency modeling, CD-GC topological reasoning, and multi-task attitude regression.

First, the input ISAR complex image $X\in C^{256\times 256}$ is uniformly divided into N non-overlapping patches, which are then flattened to obtain patch embeddings $R\in C^{N\times 1\times d\times d}$. These patches are subsequently processed by a dual-branch complex feature extraction module: the deep branch is composed of three cascaded complex convolutional layers, while the shallow branch contains a single complex convolutional layer. The outputs of the two branches are added pointwise and activated by a complex ReLU, yielding the initial complex features $F_{0}$, which encode the complete complex information. To preserve spatial positional information, learnable position encodings $E_{pos}$ are added to $F_{0}$, and the resulting representations are fed into three cascaded CD-Transformer modules. Each module builds a global receptive field through a complex self-attention mechanism, captures long-range dependencies among the patches, and outputs context-aware features $F_{1}$. The feature vectors corresponding to the patches are then treated as graph nodes V, and the spatial adjacency relationships between patches are regarded as edges ε, thus forming a graph structure G=(V,ε). On this graph, the CD-GC module explicitly perceives the topological relations of the space target under attitude variations through multiple rounds of message passing. It models the relative spatial positions of the satellite components and produces topology-enhanced features $F_{2}$. The features $F_{2}$ are globally pooled, converted to real-domain features by taking their magnitudes, and then passed to three structurally identical yet parameter-independent MLP prediction heads, which, respectively, regress the pitch angle $\theta_{p}$, the yaw angle $\theta_{y}$, and the roll angle $\theta_{r}$. The network is trained in an end-to-end manner, and the loss function is a weighted mean squared error:

(1) $$L=\lambda_{p}{\left(\theta_{p}-\overset{\wedge }{\theta_{p}}\right)}^{2}+\lambda_{y}{\left(\theta_{y}-\overset{\wedge }{\theta_{y}}\right)}^{2}+\lambda_{r}{\left(\theta_{r}-\overset{\wedge }{\theta_{r}}\right)}^{2}$$

where $\lambda_{p},\lambda_{y},\lambda_{r}$ are the weight coefficients for the respective loss terms, and $\overset{\wedge }{\theta_{p}},\overset{\wedge }{\theta_{y}},\overset{\wedge }{\theta_{r}}$ denote the ground-truth values of the pitch, yaw, and roll angles. The Adam optimizer is adopted with an initial learning rate of 10^−5^. In all experiments, we set $\lambda_{p}=\lambda_{y}=\lambda_{r}=1$, because the three attitude angles share the same numerical range [−30°, +30°] and physical scale.

### 3.2. Complex-Domain Transformer Module

**Figure 6:**
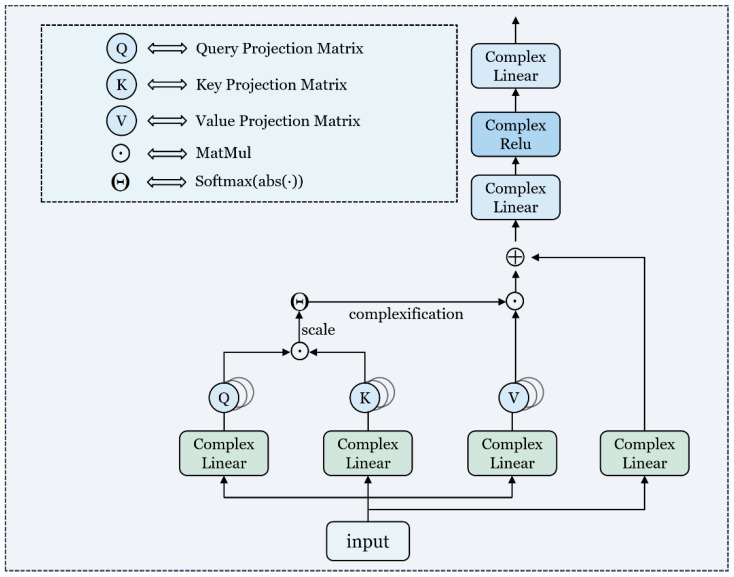
Structure of the CD-Transformer module.

The real-domain dot-product attention in the standard Transformer is inherently incompatible with complex data. The CD-Transformer module generates query, key, and value matrices through complex linear projections, preserves the complex nature of the value matrix during attention computation, and introduces a parallel fully connected branch to compensate for the information attenuation caused by complex-domain operations. The module structure is illustrated in Figure 6.

The sum of the learnable position encoding $E_{pos}$ and the feature $F_{0}$ is taken as the input, which is then projected into query Q, key K, and value V matrices through complex-domain linear projection layers. A complex linear projection layer splits the input complex vector into its real and imaginary parts and applies independent real-domain and imaginary-domain weight matrices for linear transformation, thereby achieving a mapping in the complex domain. The complex attention scores are obtained as the product of Q and the conjugate transpose of K; the modulus of these scores is normalized via softmax to obtain real-domain attention weights, which are subsequently complexified and used to weight V:

(2) $$W_{Atten}=soft\max(abs(\frac{\left|QK^{T}\right|}{\sqrt{d}})),W_{Atten}^{\prime }=W_{Atten}+j0$$

(3) $$H=W_{Atten}^{\prime }•V$$

where $K^{T}$ denotes the conjugate transpose of K, and d is the scaling factor. This approach preserves the completeness of complex features while avoiding the uncertainty of complex probabilistic normalization. To enhance the subspace representation capability, multi-head attention is adopted to improve the expressive power, and the outputs of all heads are concatenated and then integrated through the complex projection matrix $W^{o}$.

(4) $$H=Concat(head_{1},\ldots ,head_{h})W^{o}$$

However, complex features are prone to amplitude attenuation during weighted summation due to phase differences, and deep complex-domain operations may also cause feature degradation. To address this, CD-Transformer adds a parallel complex fully connected branch that projects the input into a fully connected branch feature $F_{f}$, preserving the complete complex information of the original input and compensating for the information loss after fusion with the attention output:

(5) $$F_{mid}=H+F_{f}$$

The fused feature $F_{mid}$ is subsequently passed into a complex feed-forward network (CFFN), which applies two layers of complex linear transformations interleaved with complex ReLU activations. This design jointly enhances the real and imaginary components of the complex representations and stabilizes the propagation of complex features across layers. The three CD-Transformer modules employ a progressive dimension compression strategy: the first module maintains an input and output dimensionality of 256; the second module compresses the 256-dimensional input to 128 dimensions; and the third module preserves the 128-dimensional configuration for both its input and output, yielding the final feature $F_{1}$ of the three-stage CD-Transformer architecture. This 256→256→128→128 progressive reduction gradually compresses the feature representations, which not only reduces the computational complexity of the multi-head self-attention operations but also naturally produces a hierarchy of features ranging from coarse to fine granularity. The shallow high-dimensional layers retain richer local details, while the deeper low-dimensional layers encode more abstract and semantically compact global context. Combined with residual connections, this progressive compression strategy effectively ensures training stability and promotes feature diversity, enabling the subsequent CD-GC module to operate on representations that are both compact and structurally informative.

### 3.3. Complex-Domain Graph Convolution Module

**Figure 7:**
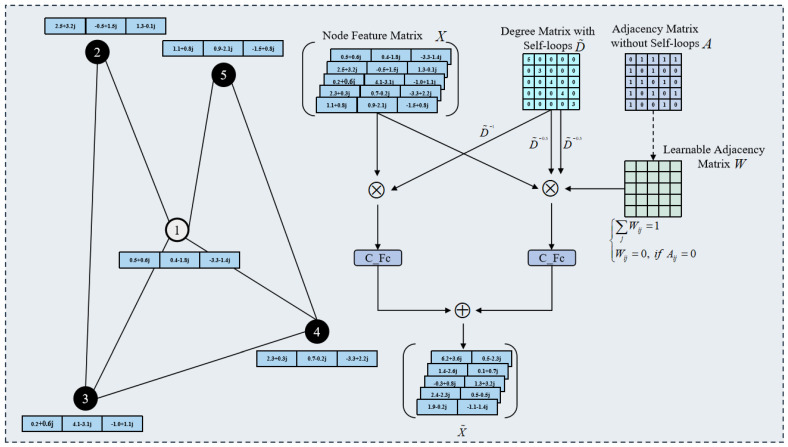
Structure of the CD-GC module.

Although CD-Transformer is capable of capturing global dependencies, it treats image patches as an unordered set and does not explicitly exploit the physical spatial relationships among space target components. Traditional graph convolutional networks are unsuitable for extracting and propagating complex-domain features; moreover, during feature updates, they apply identical operations to a node itself and its neighboring nodes, lacking discriminability and restricting the interaction between self-features and neighborhood features. To address these limitations, the CD-GC module employs a graph structure to explicitly model topological constraints and introduces learnable adjacency weights and a dual-path update mechanism, thereby enhancing the flexibility and discriminability of part-level feature interactions. The module structure is illustrated in Figure 7.

The feature vectors $F_{1}$ output by the CD-Transformer are regarded as graph nodes, forming the node feature matrix X. An 8-neighborhood adjacency relationship is constructed based on the spatial positions of the image patches in the original image, yielding the undirected adjacency matrix A. After adding self-loops, $A^{*}=A+I$, and the diagonal matrix formed by the sum of each row is the degree matrix D. The CD-GC layer uses dual information propagation paths:

Path 1 is the left-side self-node update: the node feature matrix X is inversely weighted by the degree matrix D to obtain the self-node features:

(6) $$X_{self}=D^{-1}•X$$

Path 2 is the right-side neighbor node update: the positions in A where connections exist are replaced by learnable probability values such that each row of the probability adjacency matrix sums to 1, yielding the probability adjacency matrix $\tilde{A}$ of this CD-GC layer. By training and updating $\tilde{A}$, different importance weights are assigned to each neighboring node, thereby learning the relationships among the components represented by different image patches in the target. At the same time, the originally undirected adjacency matrix becomes directed and weighted during the actual information propagation, enhancing the flexibility of the model. Its mathematical expression is shown below:

(7) $$\left\{\begin{array}{l} M=A⊙P\\ \tilde{A}={\left(\sum_{j}M_{ij}\right)}^{-1}•M \end{array}\right.$$

where ⊙ denotes the Hadamard product, P is the learnable probability value matrix, and $\sum_{j}M_{ij}$ represents the diagonal matrix formed by summing each row. In the end-to-end training, the learnable matrix P is initialized with small positive values and updated via standard backpropagation. Subsequently, normalization is performed using the probability adjacency matrix $\tilde{A}$ while retaining the degree matrix D, in order to preserve the stability of the graph structure, prevent over-smoothing, and thereby enhance the expressive power and generalization capability of the graph neural network. The matrix $D^{-1/2}\tilde{A}D^{-1/2}$, obtained from the degree matrix D and the probability adjacency matrix $\tilde{A}$, is used to update the node feature matrix X, yielding the aggregated neighbor node features $X_{neighbor}$:

(8) $$X_{neighbor}=D^{-1/2}\tilde{A}D^{-1/2}X$$

The outputs of the two paths are each passed through a complex linear transformation layer and then summed to obtain the recomposed features of the current CD-GC layer:

(9) $$\tilde{X}=D^{-1/2}\tilde{A}D^{-1/2}XW_{around}+D^{-1}XW_{main}$$

where $W_{around}$ is the complex weight matrix for transforming the aggregated neighbor node features through the corresponding complex linear transformation layer, and $W_{main}$ is the complex weight matrix for transforming the self-node features through the corresponding complex linear transformation layer. The recomposed features $\tilde{X}$ are fed into the next CD-GC layer as its input node feature matrix X. To ensure effective information interaction between the farthest node pairs under the 8-neighborhood graph structure, seven cascaded CD-GC layers are designed. A complex ReLU activation is inserted between every two adjacent layers to enhance nonlinearity. To incorporate both shallow local topology and deep global topology, the outputs of the first six layers are concatenated along the feature dimension, compressed and activated via C_Fc, and then added to the output of the seventh layer, achieving multi-scale topological feature complementarity. The final topology-enhanced features $F_{2}$ are globally average-pooled, converted to real-domain features by taking their magnitudes, and subsequently fed into three independent MLP prediction heads, which, respectively, output the estimates of the pitch, yaw, and roll angles.

## 4. Experiments

To verify the effectiveness of the proposed CD-TrGNN, four groups of experiments are conducted on the unified ISAR dataset in this section: (1) comparative experiments, which compare the performance with six representative networks, covering mainstream methods in both the real-domain and complex-domain; (2) ablation experiments, which quantify the contribution of each core module by progressively removing it; (3) robustness experiments, which evaluate the anti-interference capability of the model under different signal-to-noise ratio conditions; and (4) generalization experiments, which validate the cross-target adaptability of the model on datasets of two space targets with different structures. The experimental setup and result analysis are presented sequentially below.

### 4.1. Experimental Setup

The original complex images (512 × 512, as listed in Table 1) are center-cropped to 256 × 256 to remove empty margins and reduce computational cost. This cropping is feasible because translational motion compensation has already aligned the target centroid with the image center. Each cropped complex image corresponds to a set of known three-axis attitude angles: pitch angle $\overset{\wedge }{\theta_{p}}$, yaw angle $\overset{\wedge }{\theta_{y}}$, and roll angle $\overset{\wedge }{\theta_{r}}$. The three-axis mean absolute error is adopted as the quantitative evaluation metric. Let $\left(\overset{\wedge }{\theta_{p}^{i}},\overset{\wedge }{\theta_{y}^{i}},\overset{\wedge }{\theta_{r}^{i}}\right)$ be the ground-truth attitude angles of the i test sample, and $\left(\theta_{p}^{i},\theta_{y}^{i},\theta_{r}^{i}\right)$ be the corresponding network predictions. The single-axis mean absolute errors for pitch, yaw, and roll are, respectively, defined as:

(10) $$E_{\tau }=\frac{1}{N}\sum_{i=1}^{N}\left|\theta_{\tau }^{i}-\overset{\wedge }{\theta_{\tau }^{i}}\right|,\tau \in \left\{p,y,r\right\}$$

The three-axis mean absolute error is defined as:

(11) $$E_{avg}=\frac{1}{3N}\sum_{i=1}^{N}\left(\left|\theta_{p}^{i}-\overset{\wedge }{\theta_{p}^{i}}\right|+\left|\theta_{y}^{i}-\overset{\wedge }{\theta_{y}^{i}}\right|+\left|\theta_{r}^{i}-\overset{\wedge }{\theta_{r}^{i}}\right|\right)$$

### 4.2. Comparative Experiments

To further validate the comprehensive advantages of CD-TrGNN over existing mainstream methods, six representative networks are selected for a horizontal comparison under unified data partitioning and training conditions. These baselines cover real-domain convolutional networks, real-domain Transformers, real-domain graph neural networks, modern convolutional architectures, and complex-domain convolutional networks, thereby spanning a broad spectrum of design paradigms—from local receptive fields to global self-attention, from unstructured grid processing to explicit graph-based topological reasoning, and from magnitude-only inputs to complex-domain operations. Collectively, they represent the most widely used or cutting-edge technical routes in current ISAR-based space target attitude estimation. A direct comparison with these diverse baselines can fully reflect the improvements of the proposed method in terms of both accuracy and stability. A brief introduction to each baseline is presented in Table 2.

**Figure 8:**
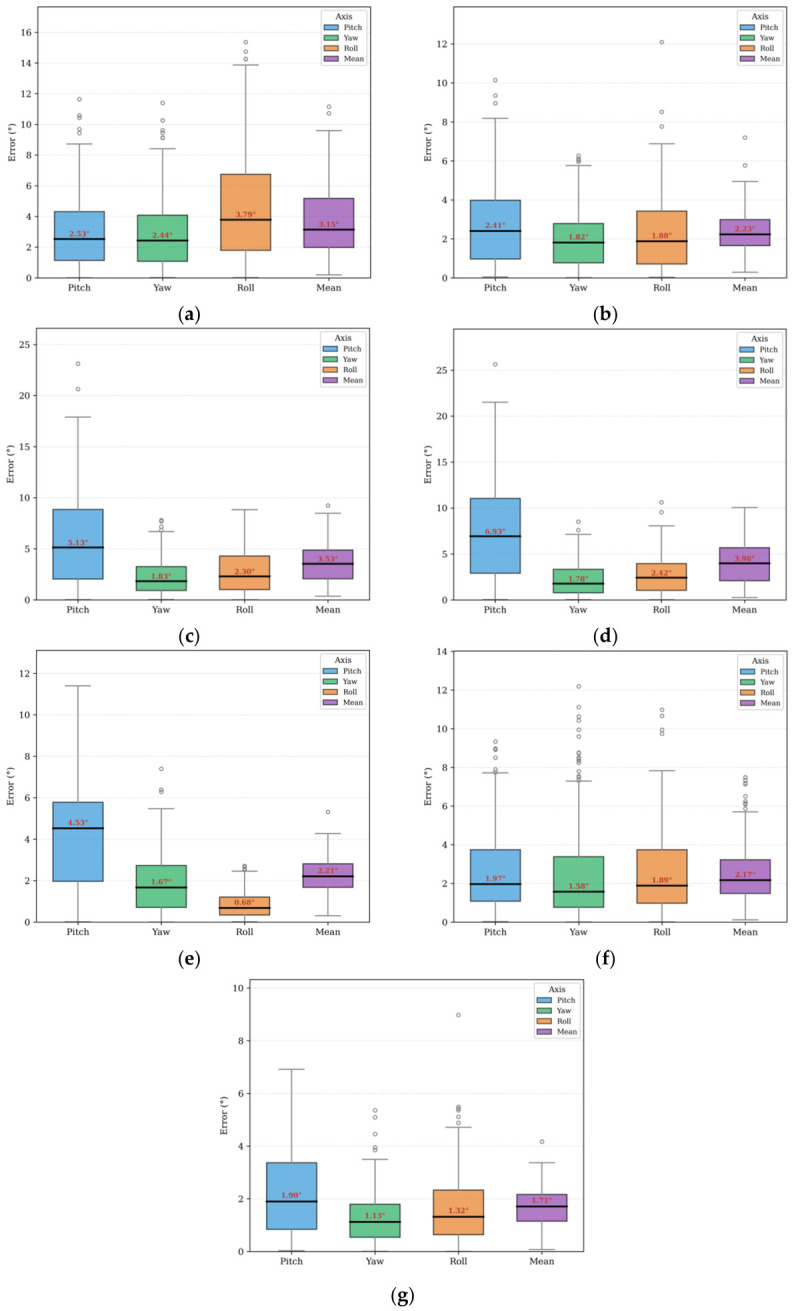
Box plots of the comparative experiments. (**a**) CNN-ResNet51; (**b**) GNN; (**c**) ViT; (**d**) ConvNeXt; (**e**) EfficientNet; (**f**) ComplexNet; (**g**) CD-TrGNN.

**Figure 9:**
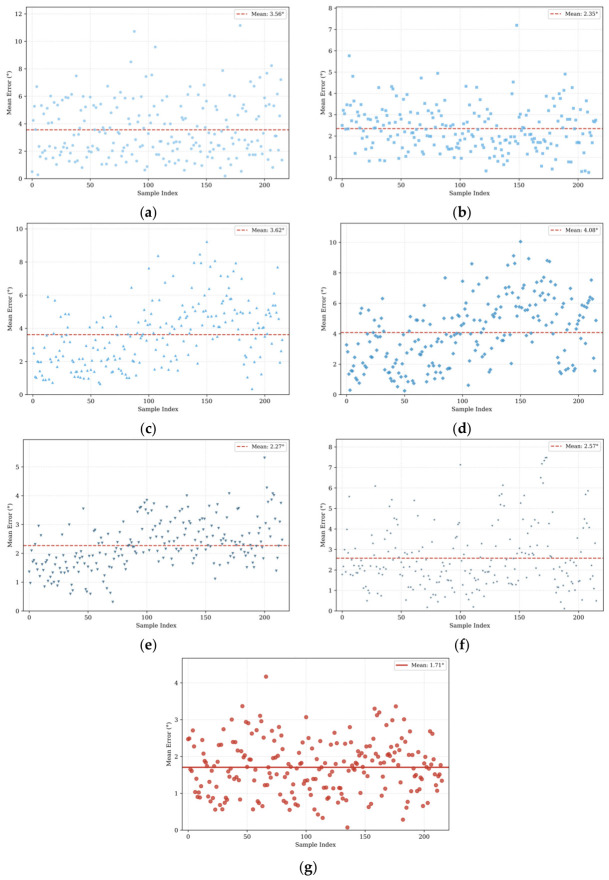
Scatter plots of the comparative experiments. (**a**) CNN-ResNet51; (**b**) GNN; (**c**) ViT; (**d**) ConvNeXt; (**e**) EfficientNet; (**f**) ComplexNet; (**g**) CD-TrGNN.

The experimental results are listed in Table 3, and the visualizations are shown in Figure 8 and Figure 9.

The experimental results show that the proposed CD-TrGNN achieves a three-axis mean error of only 1.70°, significantly outperforming all baseline methods. Compared with real-domain convolutional approaches, CNN-ResNet51 and ConvNeXt yield mean errors of 3.56° and 4.08°, respectively—more than twice that of CD-TrGNN. CD-TrGNN fully preserves the phase information of ISAR images by operating in the complex domain, avoiding the feature loss caused by magnitude-only processing. Meanwhile, the global attention of its Transformer module enables the modeling of dependencies across distant components.

In comparison with ViT, whose mean error is 3.62° and pitch error reaches as high as 5.84°, indicating unstable estimates. This instability arises because the standard ViT relies on real-domain dot-product attention, which can only process magnitude images in this setting and thus discards phase information. Moreover, ViT treats image patches as an unordered sequence, completely ignoring the spatial structural relationships among satellite components. In contrast, CD-TrGNN constructs self-attention in the complex domain and subsequently introduces explicit topological constraints through its graph convolution module, effectively remedying these deficiencies.

Compared with the graph neural network method, GNN achieves a mean error of 2.34°, outperforming both CNN and ViT. This result confirms the effectiveness of topological modeling for attitude estimation: the rigid structure of satellites makes the spatial relationships between components a reliable cue for attitude. However, the fixed 8-neighborhood graph structure and real-domain feature processing limit the expressive capability of GNN. CD-TrGNN overcomes these limitations by employing a learnable adjacency matrix that adaptively adjusts edge weights, together with a dual-path update mechanism in the complex domain, thereby further unlocking the potential of topological reasoning.

When compared with the complex-domain convolutional method ComplexNet, which achieves a mean error of 2.57°—an improvement of nearly 1° over CNN-ResNet51—the advantage of conducting operations in the complex domain over magnitude-only processing is verified. Nonetheless, ComplexNet is still restricted by the local receptive field of convolutions. CD-TrGNN inherits the benefit of complex-domain processing and further integrates Transformer-based global modeling and GNN-based topological reasoning, achieving synergistic enhancement among these three components.

These box plots further reveal the estimation stability of each method from a statistical perspective. CNN-ResNet51 and ConvNeXt exhibit wide boxes with high-end outliers, indicating severe estimation deviations at certain attitudes. GNN has a relatively compact box but a higher median, reflecting the limitations of its fixed graph structure. ViT shows an extremely wide box span in the pitch direction. In contrast, CD-TrGNN yields the most compact box with its median close to the mean, demonstrating consistently low errors across all test samples.

Figure 9 shows the per-sample mean errors of the 216 test samples via scatter plots. For ConvNeXt and ViT, the scatter distributions extend beyond 5°, and multiple high-error samples are observed. Although most of the EfficientNet samples are concentrated in the low-error region, a small number still fall within the 3–5° range. In contrast, the CD-TrGNN samples are densely clustered within the narrow range of 1.5–2.0°, confirming the estimation consistency of the model at the sample level.

### 4.3. Ablation Experiments

**Figure 10:**
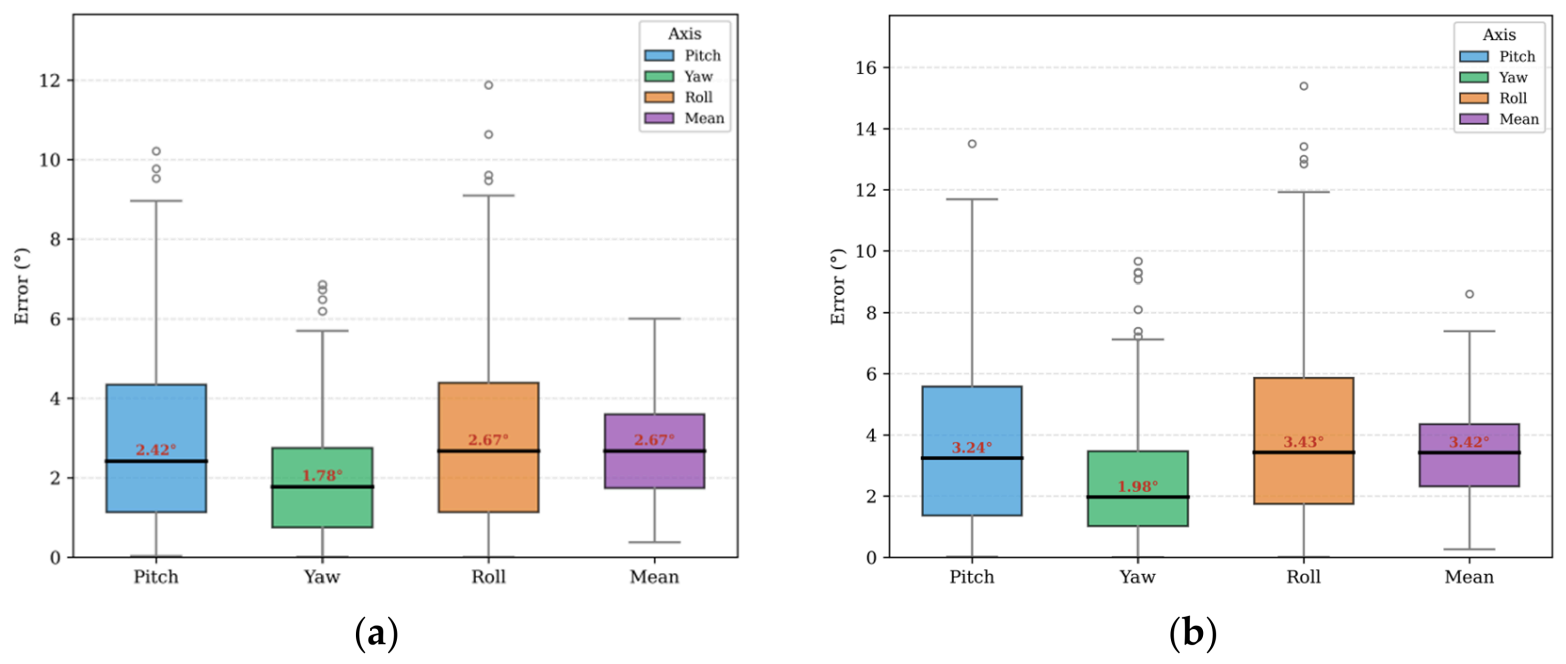

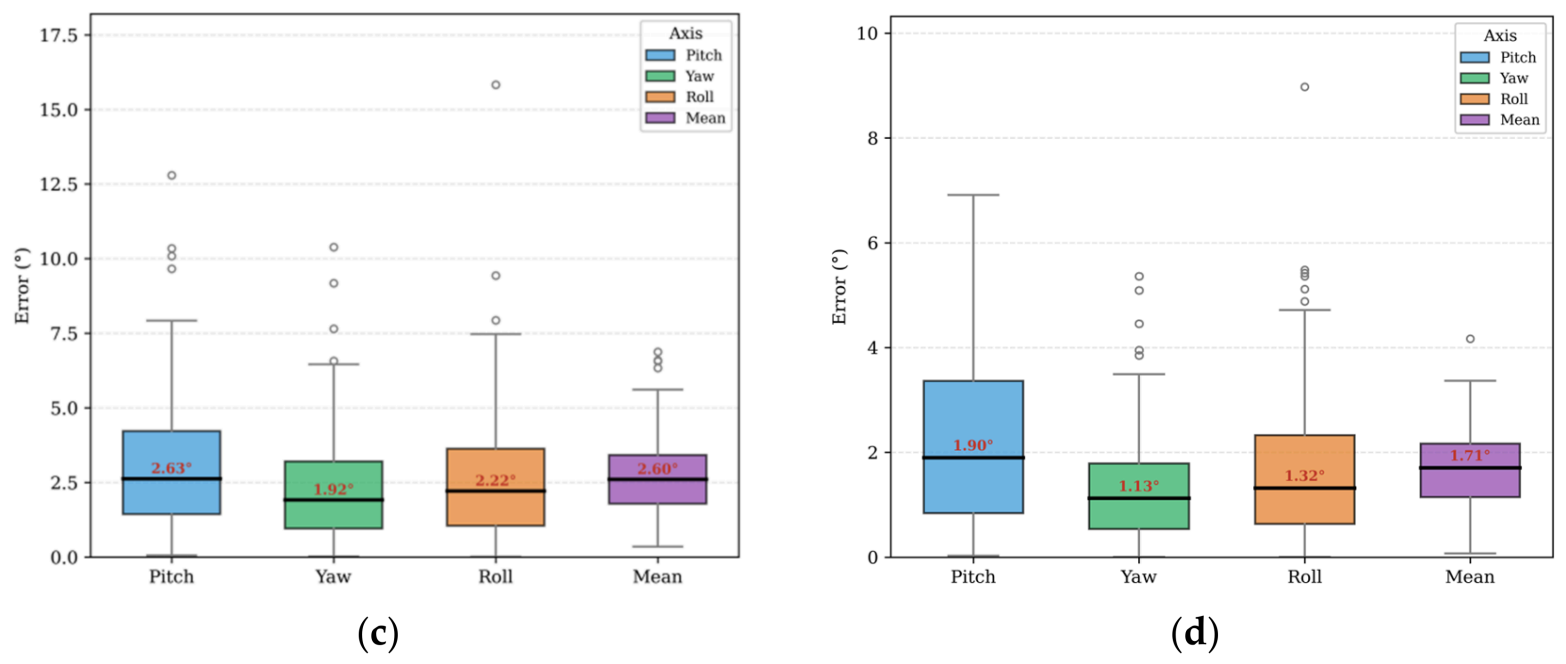
Box plots of the ablation experiments. (**a**) Real-TrGNN; (**b**) w/o Transformer; (**c**) w/o GNN; (**d**) CD-TrGNN.

**Figure 11:**
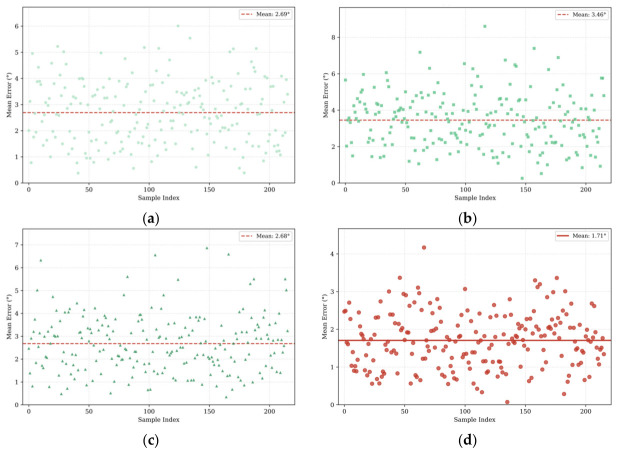
Scatter plots of the ablation experiments. (**a**) Real-TrGNN; (**b**) w/o Transformer; (**c**) w/o GNN; (**d**) CD-TrGNN.

To systematically evaluate the specific contributions of complex-domain processing, the Transformer global attention mechanism, and graph-convolution topological reasoning to attitude estimation accuracy, three structured variants are designed: one that degenerates complex operations to the real domain (Real-TrGNN), one that removes the Transformer module (w/o Transformer), and one that removes the graph convolution branch (w/o GCN). With all other network structures and training hyperparameters kept unchanged, these variants and the full CD-TrGNN are trained and tested on the same dataset. By comparing their performance differences, the accuracy gains contributed by each module can be quantified. The experimental results are presented in Table 4, and the visualization results are shown in Figure 10 and Figure 11.

The experimental results in Table 4 show that Real-TrGNN causes the mean error to rise from 1.70° to 2.69°. This indicates that complex-domain processing is not merely a change in numerical form, but a deep exploitation of the joint amplitude-phase information contained in ISAR complex images. Real-domain processing splits amplitude and phase into independent channels, thereby destroying the complete representation of electromagnetic scattering information.

w/o Transformer results in a surge of the mean error to 3.46°, representing the most severe degradation among the three variants. This variant relies solely on local complex convolutions and graph convolutions, lacks a global receptive field, and thus struggles to effectively model the spatial relationships between distant components such as solar panels and the satellite main body, leading to comprehensive deterioration in all three axes. This result validates the critical role of the complex-domain self-attention mechanism in capturing long-range dependencies.

w/o GCN raises the mean error to 2.67°. Although this variant retains the global modeling capability of the complex-domain Transformer, it treats image patches as unordered nodes and ignores the physical connections and spatial adjacency relationships among satellite components, resulting in the underutilization of topological prior information.

Figure 10 and Figure 11 present the error distributions of all variants through box plots and scatter plots, respectively. Among them, w/o Transformer exhibits the widest box span, with the interquartile ranges (IQR) in the yaw and roll directions significantly stretched and high-end outliers appearing; in its scatter plot, multiple samples exhibit errors exceeding 6°. The scatter points of Real-TrGNN and w/o GCN are mainly concentrated in the 2–4° range, but a small number of deviating samples still exist. The scatter points of the full CD-TrGNN are tightly clustered below 2°, with over 85% of the sample errors falling below 2.5°. The above ablation results collectively demonstrate that complex-domain processing, Transformer global attention, and GNN topological reasoning work synergistically and are all indispensable for ISAR complex image attitude estimation.

### 4.4. Robustness Experiments

**Figure 12:**
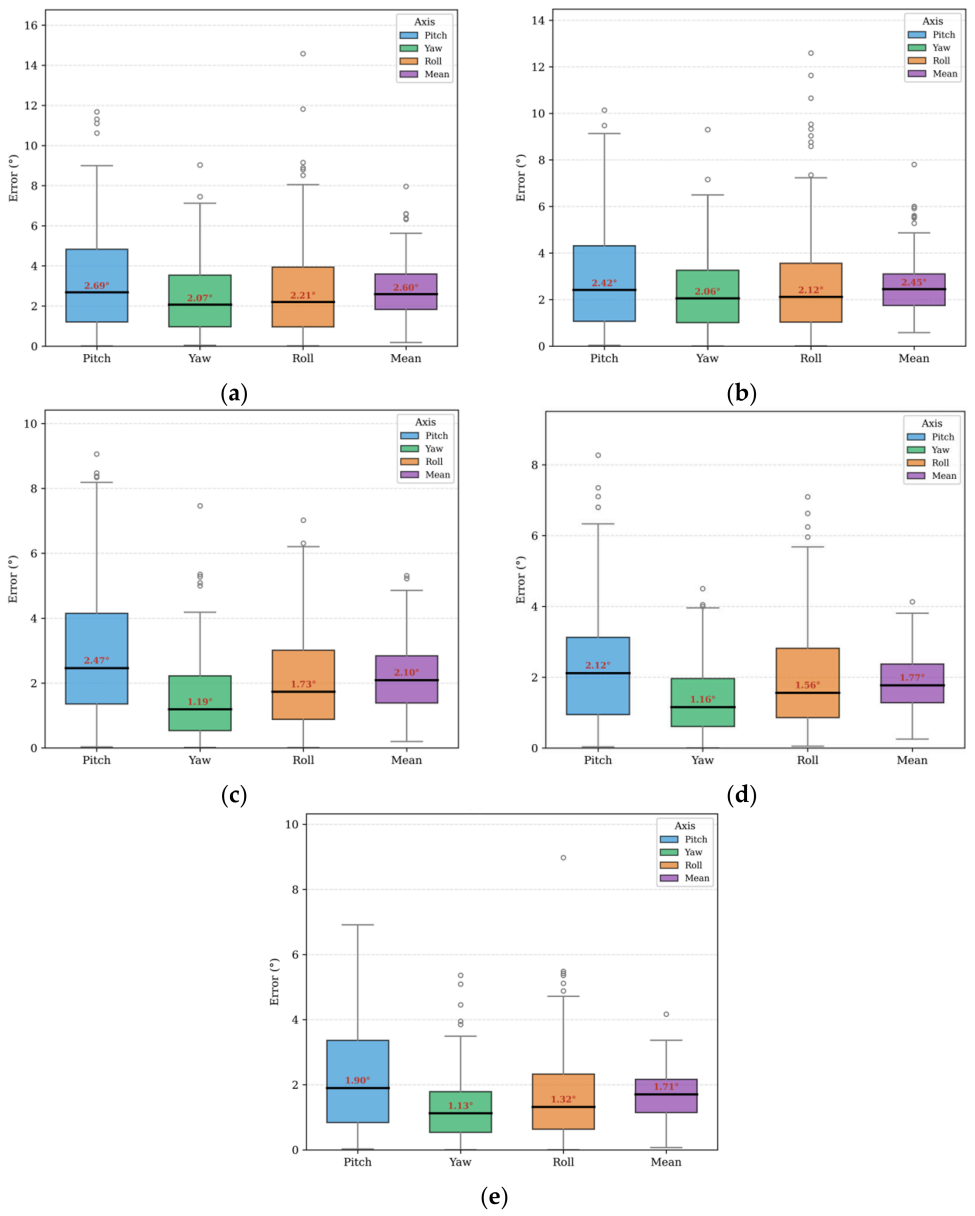
Box plots of the robustness experiments. (**a**) 5 dB; (**b**) 10 dB; (**c**) 15 dB; (**d**) 20 dB; (**e**) Clean.

**Figure 13:**
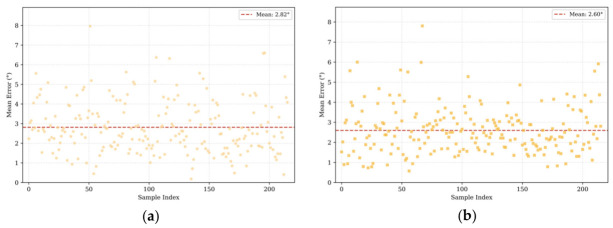

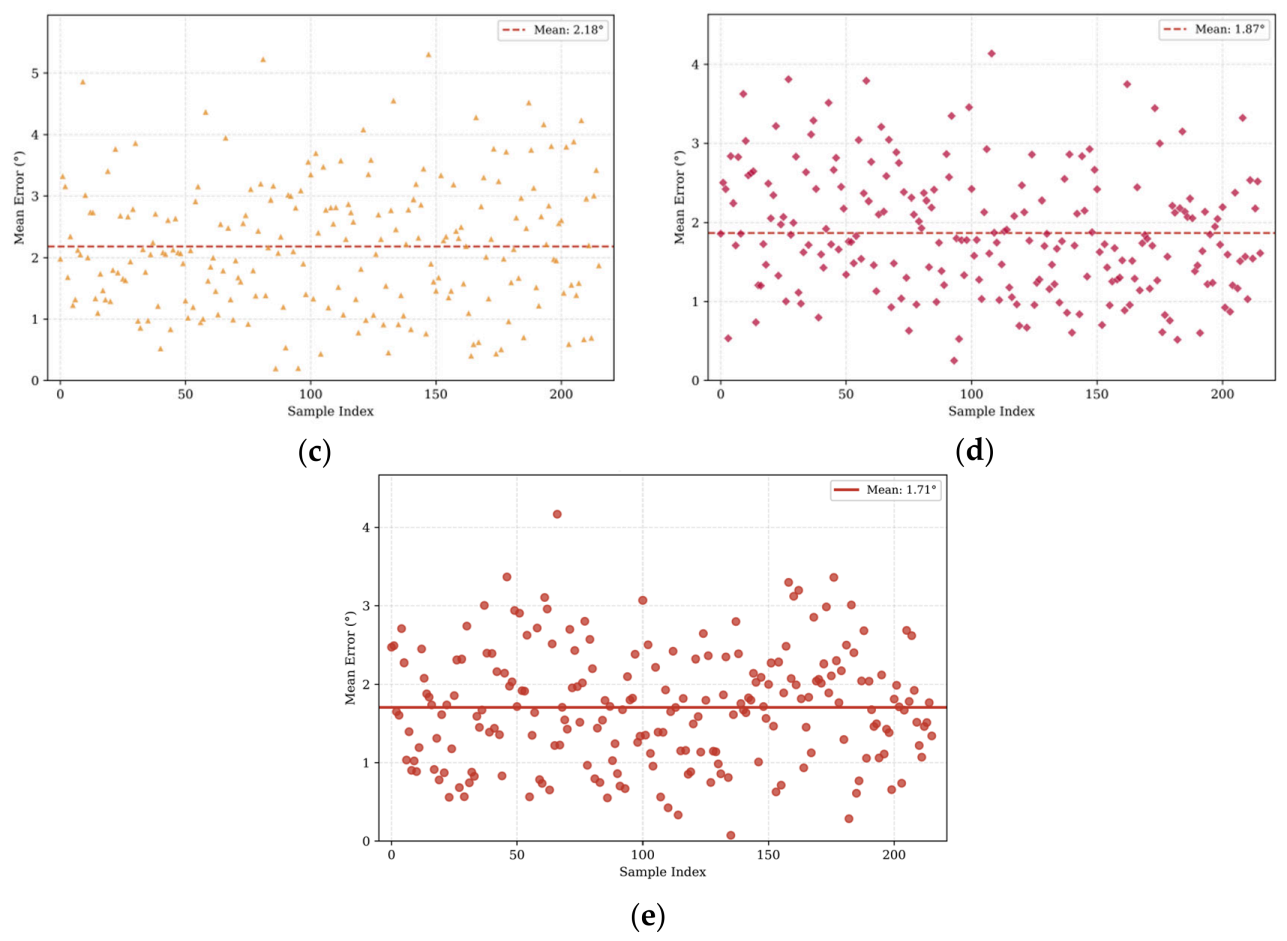
Scatter plots of the robustness experiments. (**a**) 5 dB; (**b**) 10 dB; (**c**) 15 dB; (**d**) 20 dB; (**e**) Clean.

In practice, ISAR imaging is inevitably affected by various interference sources, among which receiver thermal noise is one of the most common and unavoidable factors. This noise corrupts both the amplitude and phase components of the complex radar echoes, degrading the signal-to-noise ratio (SNR) of the resulting ISAR images. As the SNR decreases, the scattering structures of target components become increasingly obscured, making reliable attitude estimation significantly more challenging. Therefore, a practically viable attitude estimation method must maintain stable and accurate performance across a range of noise levels. To systematically evaluate the noise robustness of CD-TrGNN, we construct four degraded test sets by adding independent and identically distributed Gaussian noise to the original clean test images. The noise variance is adjusted to achieve four SNR levels: 5 dB, 10 dB, 15 dB, and 20 dB. Notably, 5 dB represents a particularly adverse condition under which only the strongest scattering centers remain visually distinguishable in the ISAR images, posing a stringent test for any estimation algorithm. Both CD-TrGNN and representative baseline methods are evaluated on these degraded datasets, and their performance degradation trends with decreasing SNR are compared. The experimental results are presented in Table 5, and the visualizations are shown in Figure 12 and Figure 13.

As the SNR decreases from the noise-free condition to 5 dB, the mean error of CD-TrGNN rises gradually from 1.70° to 2.81°, with an increase of only 1.11°, demonstrating favorable robustness against noise-induced degradation. Even under the severely contaminated 5 dB condition, where most scattering structures are heavily obscured by noise, the model maintains a mean error of 2.81°. This performance is considerably better than that of several baseline methods evaluated under noise-free conditions, such as CNN-ResNet51 at 3.56° and ViT at 3.62°, underscoring the inherent advantage of complex-domain processing. The strong noise resilience of CD-TrGNN can be attributed to two complementary mechanisms. On the one hand, the complex-domain Transformer module, empowered by its global self-attention mechanism, is able to suppress noise-corrupted regions while maintaining focus on high-confidence scattering structures that persist across the image. On the other hand, the graph convolution module enforces topological consistency across component features, acting as a structural regularizer that prevents isolated noise-induced perturbations from propagating into large estimation errors. Notably, at SNR levels of 15 dB and above, the performance of CD-TrGNN approaches that of the noise-free condition, indicating that the model possesses strong robustness against moderate-to-high intensity noise and can operate reliably in typical radar imaging environments.

The box plots in Figure 12 provide a clear visualization of how the estimation error distribution evolves as the SNR decreases. At 5 dB, the CD-TrGNN box is only slightly widened compared with the noise-free condition, the median remains well below 3°, and no significant outliers appear, indicating that the model maintains stable predictions even under severe noise. At 10 dB, the box narrows considerably, and the interquartile range shrinks toward the noise-free level. At 15 dB and 20 dB, the box height becomes nearly indistinguishable from that of the clean condition, suggesting that the effect of noise on the estimation error is largely negligible at moderate SNR levels and above. The scatter plots in Figure 13 further corroborate this trend at the sample level. Under the 5 dB condition, although the error distribution is visibly more dispersed than in cleaner cases, most sample errors remain within 4.5°, and no catastrophic outliers are observed. As the SNR improves to 20 dB, the scattered points become tightly clustered below 2°, exhibiting a distribution that overlaps almost completely with that of the noise-free case. The above results consistently indicate that CD-TrGNN possesses strong intrinsic robustness to Gaussian complex noise and has the potential to deliver reliable attitude estimates in typical radar imaging environments, where moderate SNR fluctuations are common.

### 4.5. Performance Evaluation on Different Target Datasets

**Figure 14:**
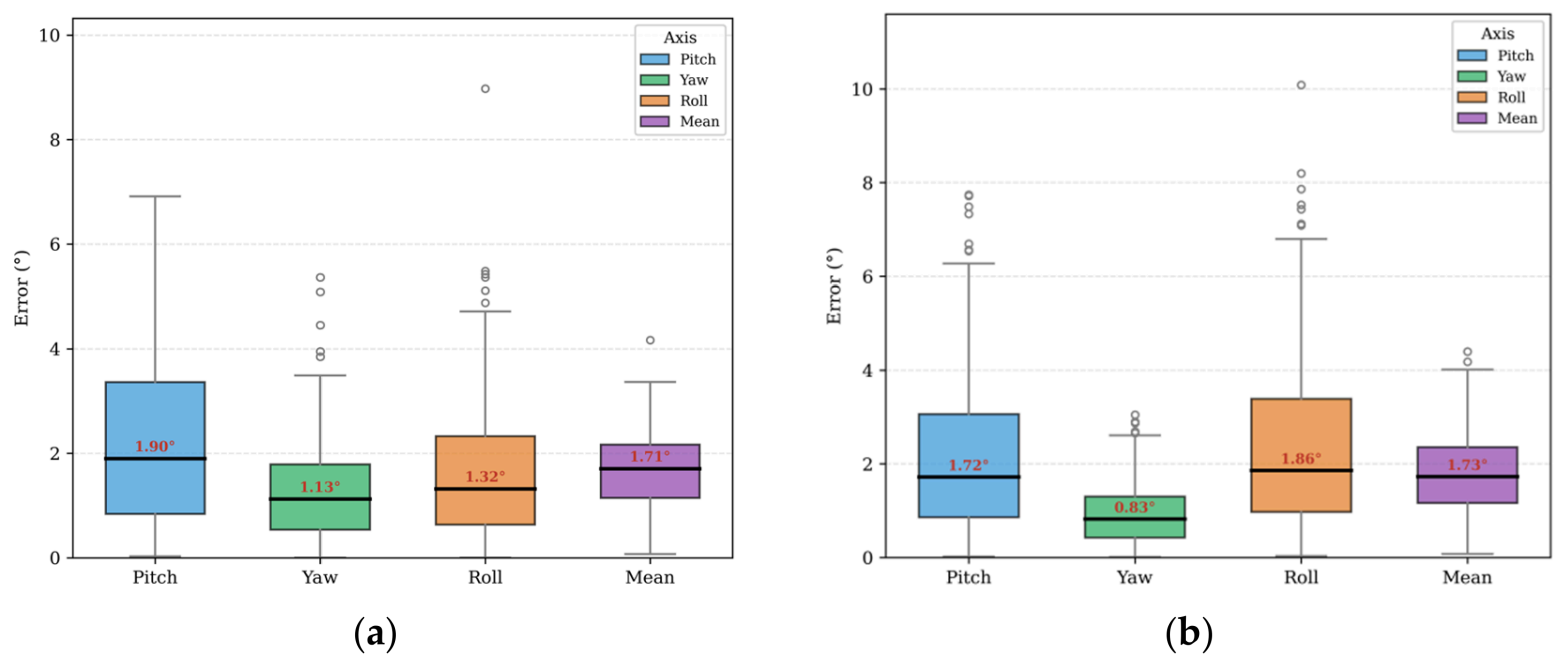
Box plots of the generalization experiments. (**a**) Target A; (**b**) Target B.

**Figure 15:**
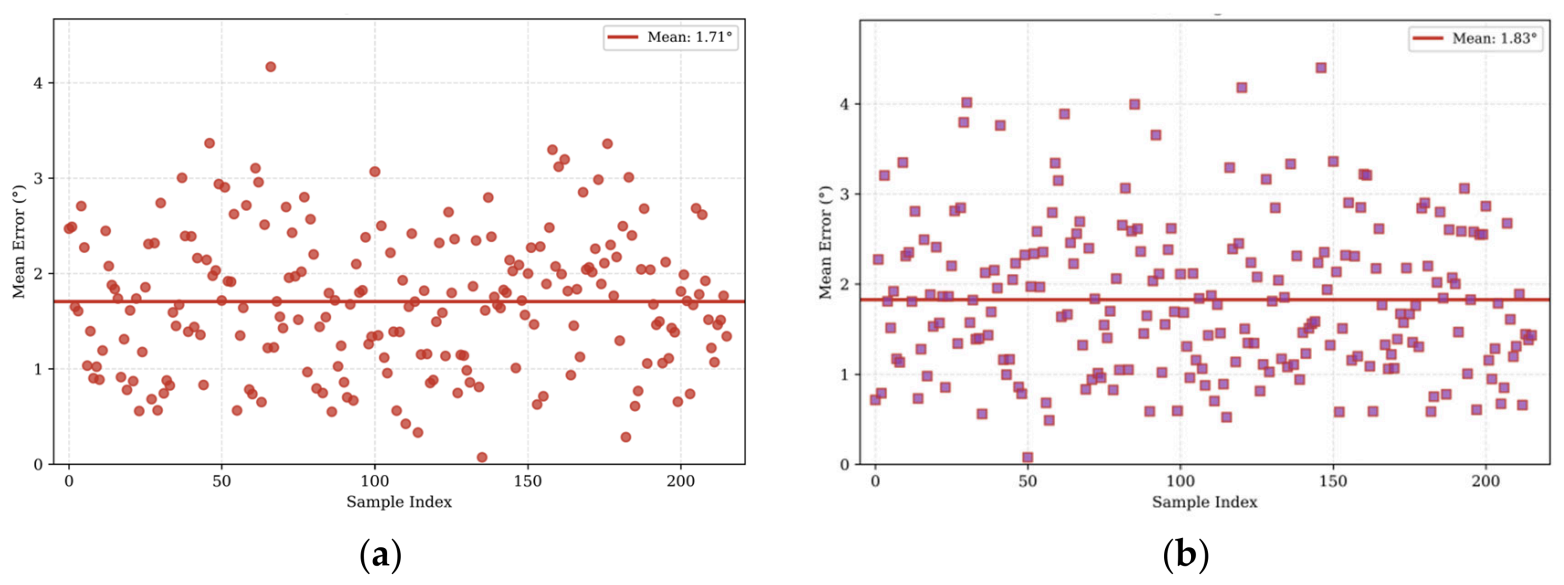
Scatter plots of the generalization experiments. (**a**)Target A; (**b**) Target B.

To evaluate the generalization capability of CD-TrGNN across satellites with different structures, independent training and testing are conducted on the ISAR complex image datasets of Target A and Target B, respectively. The attitude angle partition rules for both datasets are identical, and the model is trained from scratch on each dataset with the same hyperparameter settings. The experimental results are listed in Table 6, and the visualizations are shown in Figure 14 and Figure 15.

The model achieves a mean error of 1.70° on Target A and 1.83° on Target B. The two results are close, with a difference of only 0.13°, indicating that the model is insensitive to variations in target geometry and possesses favorable cross-target generalization capability.

Examining individual axes, Target B achieves notably good accuracy in yaw estimation. This may be attributed to the more regular scattering projection structure of Target B in the yaw direction: the more compact main body of Target B results in clearer patterns of ISAR image feature variations induced by yaw rotation, making it easier for the model to capture the attitude variation characteristics. However, the roll error of Target B is noticeably higher than that of Target A. The probable reason is that the compact structure of Target B leads to more drastic changes in the distribution of equivalent scattering centers under roll attitude variations, together with the lack of stable long-distance feature references provided by large solar panels, thereby increasing the regression difficulty.

Figure 14 presents the distribution details of per-sample mean errors on the two target datasets via box plots. For Target A, the boxes of all three axes are compact, the medians are close to the means, and the IQRs are all within 1.2°, indicating that the model yields stable estimates for Target A. For Target B, the yaw box is narrower, with an IQR of approximately 0.8°, whereas the roll box is wider, with an IQR of approximately 1.8°, which is consistent with the higher roll error observed.

Figure 15 shows the error distributions of the 216 test samples for both targets via scatter plots. The distributions of the two datasets exhibit similar shapes, both concentrated within the 1.5–2.5° range, with no significant outliers or deviating samples. Although the mean error of Target B is slightly higher than that of Target A, the sample errors are essentially within 4°, and over 85% of the sample errors are below 2.5°.

### 4.6. Experiment Summary

This section presents a systematic and comprehensive experimental validation of the proposed CD-TrGNN on the ISAR complex image dataset. The comparative experiments demonstrate that CD-TrGNN achieves a three-axis mean error of 1.70°, significantly outperforming existing baseline methods in both the real domain and complex domain. The box plots and scatter plots, respectively, confirm its dual advantages in accuracy and stability from the perspectives of statistical distribution and sample-level granularity. The ablation experiments quantitatively verify the irreplaceable contributions of complex-domain processing, the global attention mechanism, and the topological reasoning module: complex operations avoid the loss of phase information; the complex-domain Transformer module resolves the limitation of long-range dependency modeling; and the graph convolution module explicitly exploits the physical structural constraints among satellite components. The distribution differences in the ablation variants in the box plots and scatter plots intuitively illustrate the failure mode when each component is removed. The robustness experiments show that even under the low-SNR condition of 5 dB, the model maintains a mean error of 2.81°, with most sample errors within 4.5°, exhibiting excellent anti-interference capability that significantly surpasses the baseline methods. The generalization experiments further confirm that CD-TrGNN can maintain stable high-accuracy estimation on two space targets with different structures, demonstrating favorable cross-target adaptability and transferability. The results of the above four groups of experiments consistently validate the advancement, robustness, and generalization capability of CD-TrGNN for ISAR-based space target attitude estimation. Notably, the depth of the CD-GC module is NOT an arbitrary choice. Since the graph connects patches via an 8-neighborhood pattern on an 8 × 8 grid, the Chebyshev distance between the farthest nodes is 7; consequently, seven graph convolution layers are required to allow full-graph information exchange. In contrast, the CD-Transformer and C-Conv layers can be flexibly stacked according to the input resolution and desired accuracy. Our ablation study already proves the necessity of each module, and the current configuration (3 Transformer blocks, 7 GC layers) achieves optimal performance for the standard 256 × 256 ISAR input, while the architecture can be readily scaled up or down with predictable effects.

## 5. Conclusions

To address the insufficient exploitation of phase information, limited global dependency modeling, and the absence of part-topology priors in ISAR-based space target attitude estimation, this paper proposes CD-TrGNN, a framework that integrates global attention and adaptive topological reasoning entirely within the complex domain to achieve direct three-axis attitude regression. The main contributions include: constructing ISAR complex image datasets for two distinct target types; designing a four-stage architecture composed of complex convolution, CD-Transformer, CD-GC, and multi-task regression heads, in which complex self-attention and learnable adjacency matrices jointly preserve phase information and explicitly encode part-level structural topology; and conducting a systematic validation through comparative, ablation, robustness, and generalization experiments. The results demonstrate that CD-TrGNN achieves a three-axis mean absolute error of only 1.70°, outperforming the best baseline by approximately 25%, while maintaining an error of 2.81° at 5 dB SNR and sub-2° accuracy on both target types. Future work will incorporate real measured data, temporal modeling, scattering-center-based semantic graphs, and few-shot transfer learning across more diverse satellite structures to further enhance practical applicability and generalization capability.

It should be noted that in the range-Doppler imaging process, after translational motion compensation (envelope alignment and autofocus), the target’s centroid is normally centered at the image origin. Therefore, the ISAR images used in this study all contain the target near the image center. CD-TrGNN relies on global self-attention and an 8-neighborhood graph structure, which are inherently robust to small residual centering errors. If the target deviates significantly from the center due to a severe motion-compensation failure, the issue lies in the preprocessing stage rather than the network itself; as a safety redundancy, random shift augmentation can be introduced during training to further improve translation invariance, which will be explored in future work.

## Figures and Tables

**Table 1:** Main parameters of the ISAR system.

Parameter	Value
Bandwidth	2 GHz
Center frequency	14 GHz
Image size	512 × 512
Pulse repetition frequency	80 Hz

**Table 2:** Introduction of Baseline Methods.

Baseline Method	Description
CNN-ResNet51 [37]	Based on ResNet50, with the input layer receiving magnitude images and the regression head outputting three-axis attitude angles.
GNN [38]	A graph structure is constructed after CNN feature extraction; a standard GCN performs topological reasoning, followed by a regression head.
ViT [39]	Utilizes the ViT-Base architecture, taking patch sequences of magnitude images as input and modeling global dependencies with real-domain self-attention.
ConvNeXt [40]	A representative modern convolutional architecture, adopting ConvNeXt-Tiny with magnitude images as input.
EfficientNet [41]	A representative efficient convolutional architecture, adopting EfficientNet-B0 with magnitude images as input.
ComplexNet [32]	A complex-domain convolutional network in which all convolutions, batch normalization, and activations are performed in the complex domain.

**Table 3:** Comparison of attitude estimation errors with baseline methods.

Method	Pitch Error	Yaw Error	Roll Error	Mean Error
CNN-ResNet51	3.00°	2.98°	4.7°	3.56°
GNN	2.75°	1.95°	2.33°	2.34°
ViT	5.84°	2.13°	2.88°	3.62°
ConvNeXt	7.26°	2.19°	2.79°	4.08°
EfficientNet	4.05°	1.91°	0.84°	2.27°
ComplexNet	2.27°	2.55°	3.48°	2.57°
**CD-TrGNN**	**2.16°**	**1.27°**	**1.67°**	**1.70°**

**Table 4:** Ablation experiment results.

Method	Pitch Error	Yaw Error	Roll Error	Mean Error
Real-TrGNN	2.97°	1.95°	3.15°	2.69°
w/o Transformer	3.80°	2.52°	4.03°	3.46°
w/o GCN	3.15°	2.26°	2.62°	2.67°
**CD-TrGNN**	**2.16°**	**1.27°**	**1.67°**	**1.70°**

**Table 5:** Robustness experiment results.

SNR	Pitch Error	Yaw Error	Roll Error	Mean Error
5 dB	3.21°	2.41°	2.81°	2.81°
10 dB	2.91°	2.26°	2.61°	2.60°
15 dB	2.89°	1.5°	2.14°	2.18°
20 dB	2.29°	1.35°	1.97°	1.87°
**Clean**	**2.16°**	**1.27°**	**1.67°**	**1.70°**

**Table 6:** Generalization experiment results.

Dataset	Pitch Error	Yaw Error	Roll Error	Mean Error
Target A	2.16°	1.27°	1.67°	1.70°
Target B	2.19°	0.92°	2.36°	1.83°

## Data Availability

The original contributions presented in the study are included in the article; further inquiries can be directed to the corresponding author.
